# Nomograms predicting cancer-specific survival for stage IV colorectal cancer with synchronous lung metastases

**DOI:** 10.1038/s41598-022-18258-w

**Published:** 2022-08-17

**Authors:** Pu Cheng, Haipeng Chen, Fei Huang, Jiyun Li, Hengchang Liu, Zhaoxu Zheng, Zhao Lu

**Affiliations:** 1 Department of Colorectal Surgery, National Cancer Center/National Clinical Research Center for Cancer/Cancer Hospital, Chinese Academy of Medical Sciences and Peking Union Medical College, Beijing, China; 2 Department of Gastrointestinal Surgery, Zhongnan Hospital of Wuhan University, Wuhan, Hubei China

**Keywords:** Metastasis, Colon cancer

## Abstract

This study aimed to establish a nomogram for the prediction of cancer-specific survival (CSS) of CRC patients with synchronous LM. The final prognostic nomogram based on prognostic factors was evaluated by concordance index (C-index), time-dependent receiver operating characteristic curves, and calibration curves. In the training and validation groups, the C-index for the nomogram was 0.648 and 0.638, and the AUC was 0.793 and 0.785, respectively. The high quality of the calibration curves in the nomogram models for CSS at 1-, 3-, and 5-year was observed. The nomogram model provided a conventional and useful tool to evaluate the 1-, 3-, and 5-year CSS of CRC patients with synchronous LM.

## Introduction

Colorectal cancer (CRC) is the second leading cause of cancer-related deaths worldwide^1^. In China, CRC is the third most common malignancy in both men and women^2^. Approximately 25% of CRC patients were reported to have metastases at the time of diagnosis^3^. Actually, metastasis was one of the leading causes of the poor prognosis of CRC patients, the survival was approximately 29 to 30 months with the current treatment modality^4^. Therefore, increasing recognization for CRC with metastasis was of crucial importance for improving patients’ survival. Regretfully, the large-scale prospective study about clinicopathological characteristics and prognostic factors for metastatic CRC were still deficient^5^.

Currently, the liver and lung are the most common sites of CRC metastases^6,7^. The lungs are second only to the liver as the site of metastasis, a recent study demonstrated that the rate of lung metastasis (LM) was present in approximately 29% of metastatic cases^8^. The incidence of LM in patients with CRC was relatively low but was frequently associated with a poor prognosis^9^. Due to the widespread use of chest CT scans in recent years, the rate of diagnosis of LM for patients with CRC increased when patients were diagnosed with CRC^10^. Therefore, it is necessary to evaluate the prognosis of CRC with synchronous LM. However, the prognosis of patients with CRC with synchronous LM has rarely been investigated. Furthermore, due to the relatively low incidence, no independent prognostic factors associated with survival in CRC patients with synchronous LM have been identified. Currently, the survival of patients with CRC is usually predicted using the TNM staging system^11^, which has limitations. A study^12^ has reported that there are differences involving the metastatic pattern between different metastasis lesions; therefore, the TNM staging system as a general evaluation tool cannot accurately predict the prognosis of CRC patients with synchronous LM, and a more individual prediction model for the survival of CRC with synchronous LM is urgently needed. The nomogram, a simple and useful tool that has been shown to provide more precise survival prediction compared to traditional TNM staging systems, and is built using independent prognostic factors^13^, has been widely used in medical research and clinical practice^14–16^.

In this study, our objective was to investigate predictive and prognostic factors of CRC with synchronous LM based on the Surveillance, Epidemiology, and End Results (SEER) database, which contains a large number of patients and their detailed clinical information, and then a nomogram was developed based on independent prognostic factors to predict 1-,3-,5-year cancer-specific survival (CSS) for CRC with synchronous LM. The nomogram was then used to stratify patients into high- and low-risk groups.

## Materials and methods

### Data sources

Patients with CRC diagnosed with synchronous LM were extracted from the SEER database between January 2010 and December 2015. The SEER is an openly accessed database, which collects cancer information on patients from 18 separate cancer registries across the United States and covers about one-quarter of the population of the whole country^17^. A patient consent form was not required for data from the SEER database as all data were de-identified prior to release and did not contain personally identifiable information from patients. This study was approved by the Ethics Committee of the National Cancer Center/National Clinical Research Center for Cancer/Cancer Hospital, Chinese Academy of Medical Sciences.

### Diagnostic and exclusion criteria

The main diagnostic criteria for this study were as follows: (a) patients with CRC; (b) stage IV patients with synchronous LM; and (c) patient’s death attributable to CRC. The exclusion criteria were patients with incomplete clinical data or pathological information. All enrolled patients were staged according to the 6th AJCC TNM staging system.

### Patient population

**Figure 1:**
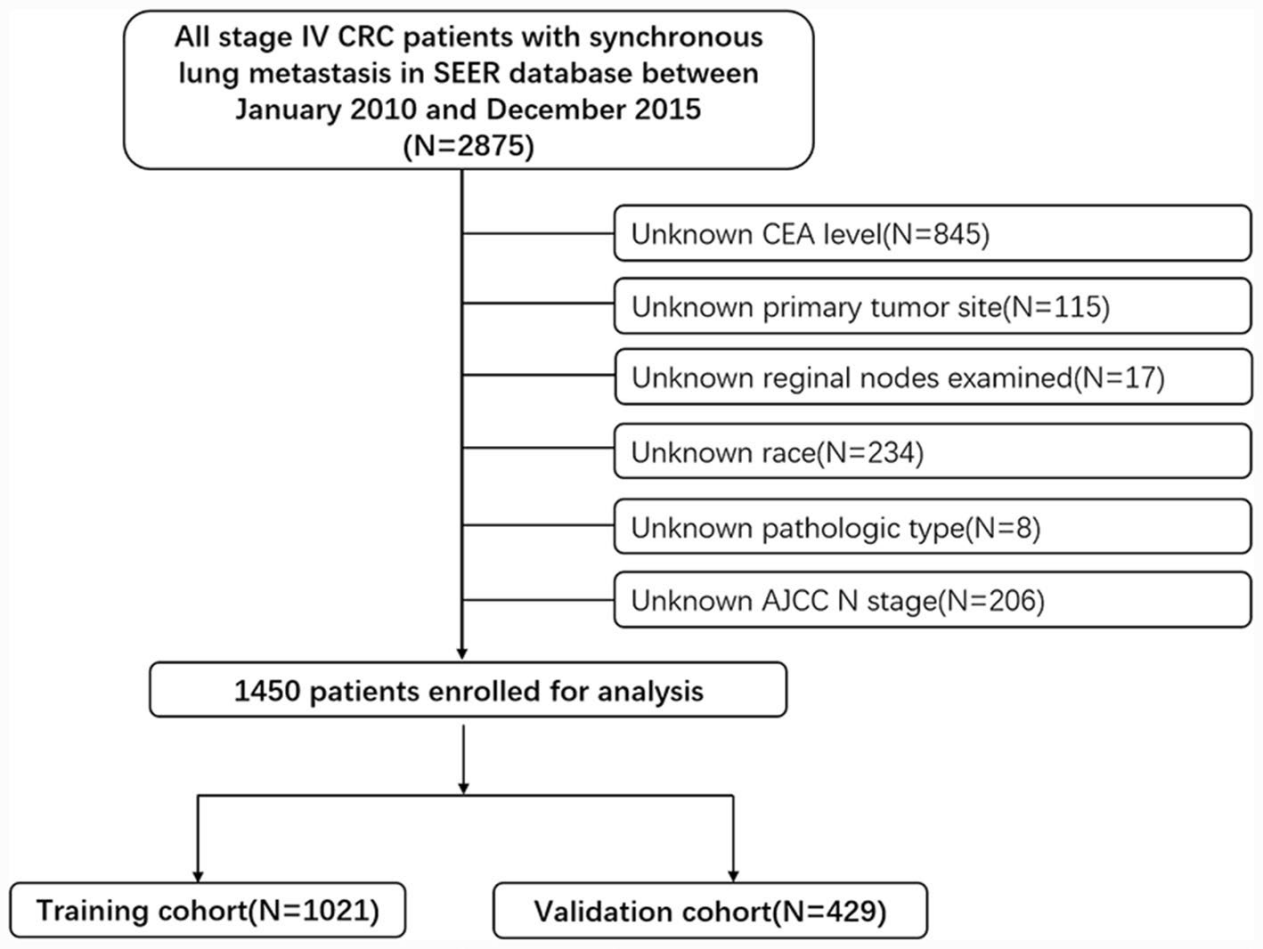
Analytical cohort and exclusion criteria of CRC patients with synchronous LM.

The patient data entered in the database were considered to be representative of the overall population. SEER*Stat version 8.3.9 was used to generate a case list. Data from SEER was used to identify patients with CRC diagnosed between 2010 and 2015, and 2875 stage IV CRC patients with synchronous lung metastasis were extracted according to related indications in SEER (“SEER Combined Mets at DX-lung (2010 +): YES”, “SEER other cause of death classification: Alive or dead due to cancer”, “ICD-O-3: 8140, 8210, 8220, 8261, 8263, 8480, 8481 and 8490”). Then, we excluded the unknown CEA level(*n* = 845), unknown primary tumor site(*n* = 115), unknown regional nodes examined(*n* = 17), unknown race(*n* = 234), unknown pathologic type(*n* = 8), unknown AJCC N stage(*n* = 206). Finally, a total of 1450 patients were enrolled in this study, which was divided into the training cohort and the validation cohort in a 7: 3 ratio by EXCEL using the Rand function. A general description of our study design is presented in Fig. 1. All CRC patients included in this study were definitively diagnosed by pathological examination and LM was diagnosed by imaging examination or pathological examination. Several clinical and tumor-related variables were collected to analyze prognosis, including age, AJCC N stage, CEA level, extra-LM (defined as involving bone, brain, and liver), primary tumor site, primary tumor size, and regional nodes examined, sex, year of diagnosis, race, pathological type, and primary tumor resection (primary CRC surgery combined with lung metastasectomy) in the SEER database.

### The construction of the CSS nomogram

The CSS was the endpoint in the present study, it was calculated from diagnosis to death of the patient or the date still alive at the last censored follow-up. CSS was assessed using the Kaplan–Meier method, with the log-rank tests used in univariate analysis. The final independent prognostic factors were identified by multivariate analysis using the Cox regression model. The nomogram was developed based on these prognostic factors (*P* < 0.05) to predict the CSS of CRC patients with synchronous LM.

### Evaluation of nomogram performance

The discrimination ability of the nomogram was evaluated using the concordance index (C-index) and AUC value^18^. AUC-index of 0.5 indicated a random chance and 1.0 indicated a perfect ability to correctly discriminate the outcome with the model. AUC values of 0.5–0.7, 0.7–0.85, and 0.85–0.95 were defined as low, middle, and high credibility, respectively^18^. The calibration ability of the nomogram was evaluated with calibration curves for 1-, 3-, and 5-year CSS comparing the predicted survival with the observed survival. The 1-, 3-, and 5-year ROC curves were used to evaluate the predictive accuracy of the nomogram for different periods. Internal validation of the nomogram was achieved with the bootstrap resampling strategy (1000 resamples). External validation was conducted in the validation cohort. Briefly, the validation cohort was individually given a risk score calculated with the nomogram equation.

### Statistical analysis

The Rand function of EXCEL was used for the randomization of patients.The Chi-square test was used to compare the differences between the training and validation cohorts for the categorical variables. The R statistical packages “rms”, “survival”, “Hmisc”, “lattice”, “Formula”, “ggplot2”, “foreign”, “nomogramFormula”, “survivalROC”, “pROC” and “timeROC” were used to calculate the C-index, plot the calibration and ROC curves, build a nomogram, and draw the Kaplan–Meier curves. SPSS23.0 (SPSS Inc., Chicago, IL) and R (R version 4.1.1, http://www.r-project.org) were used for statistical analysis, and a *P*-value < 0.05 was considered statistically significant. This study has been reported in line with the TRIPOD statement^19^.

### Ethics approval

The studies involving human participants were reviewed and approved by the Ethics Committee of the National Cancer Center/National Clinical Research Center for Cancer/Cancer Hospital, Chinese Academy of Medical Sciences. The written consent form was not required for data from the SEER database as all data were de-identified prior to release and did not contain personally identifiable information from patients.

### Methods statement

The authors confirm that all methods were carried out in accordance with relevant guidelines and regulations as publicly available data is used.

## Results

### Patient characteristics

From 2010 to 2015, 74,926 CRC patients were registered in the SEER database, a total of 1450 CRC patients with synchronous LM were included in our study, and patients were assigned in a nearly7:3 ratio to the training cohort and the validation cohort randomly. A total of 1021 patients with complete information were included in the final analysis for the training cohort. For the validation cohort, a total of 429 patients were included in the final analysis after the application of the same inclusion and exclusion criteria. The clinicopathological characteristics and demographics of the entire cohort (*n* = 1450), including the training (*n* = 1021), and validation (*n* = 429) subsets are described in (Table 1). In the training and the validation cohort, the majority of patients were ≥ 60 years old at diagnosis (62.1 and 64.3%, respectively). Most patients had an adenocarcinoma histological type (86.2 and 86.9%, respectively), and most patients’ tumors were located in the descending colon (45.9 and 45.7%, respectively). Extra-LM were identified in 73.8% and 76.2% of the patients in the training and validation cohorts, respectively, and the CEA levels were positive in 84.6% and 88.1% of the patients. Whites accounted for 81.9% and 80.4% of all cases, respectively. Most people were AJCC N0 (39.3 and 41.7%, respectively) and N1 (40.5 and 40.1%, respectively). Across the study population of the two cohorts, only 0.8% and 0.7% of the patients underwent primary surgery, and 62.6% and 65.7% of the patients had no regional nodes examined. The primary tumor size of most patients was < 3 cm (59.6% and 62.0%, respectively). The range of CSS ranged from 0–106 months and 0–107 months in both cohorts, respectively. The mean CSS was 20.35 and 19.84 months. with a median CSS of 14 months and 15 months, respectively. Most of the variables including median survival month (*P* = 0.739), number of events (*P* = 0.102), age (*P* = 0.421), year of diagnosis (*P* = 0.182), race (*P* = 0.514), primary tumor site (*P* = 0.938), pathological type (*P* = 0.742), primary tumor resection (*P* = 1.000), Extra-lung metastasis (*P* = 0.343), CEA(*P* = 0.084), regional nodes examined (*P* = 0.723), AJCC N stage (*P* = 0.582), primary tumor size (*P* = 0.683) showed no significant differences between the training and the validation cohort, which indicated that patients in the training and validation cohorts had a balanced survival distribution and baseline clinical characteristics.

**Table 1:** The clinicopathologic characteristics of patients in the training and validation cohorts.

Variable	Entire cohort (*N* = 1450)		Training cohort (*N* = 1021)		Validation cohort (*N* = 429)		*P-*value
	No. of Patients	%	No. of Patients	%	No. of Patients	%	
**Survival months**							0.739
Median	14	14	15				
Range	0–107	0–106	0–107				
**Number of events**							0.102
Alive	151	10.4	115	11.3	36	8.4	
Dead	1299	89.6	906	88.7	393	91.6	
**Sex**							0.031
Male	773	53.3	563	55.1	210	49.0	
Female	677	46.7	458	44.9	219	51.0	
**Age, years**							0.421
< 60	540	37.2	387	37.9	153	35.7	
≥ 60	910	62.8	634	62.1	276	64.3	
**Year of diagnosis**							0.182
2010	173	11.9	118	11.6	55	12.8	
2011	239	16.5	172	16.8	67	15.6	
2012	243	16.8	172	16.8	71	16.6	
2013	264	18.2	180	17.6	84	19.6	
2014	252	17.4	167	16.4	85	19.8	
2015	279	19.2	212	20.8	67	15.6	
**Race**							0.514
Black	269	18.6	185	18.1	84	19.6	
White	1181	81.4	836	81.9	345	80.4	
**Primary tumor site**							0.938
Transverse colon	76	5.2	54	5.3	22	5.1	
Ascending colon	254	17.5	182	17.8	72	16.8	
Descending colon*	665	45.9	469	45.9	196	45.7	
Rectum	455	31.4	316	31.0	139	32.4	
**Pathological type**							0.742
Adenocarcinoma in adenoma	130	9.0	93	9.1	37	8.6	
Adenocarcinoma	1253	86.4	880	86.2	373	86.9	
Mucinous adenocarcinoma	59	4.1	41	4.0	18	4.2	
Signet–ring cell carcinoma	8	0.6	7	0.7	1	0.2	
**Primary tumor resection**							1
Yes	11	0.8	8	0.8	3	0.7	
No	1439	99.2	1013	99.2	426	99.3	
Extra–lung metastasis*							0.343
Yes	1081	74.6	754	73.8	327	76.2	
No	369	25.4	267	26.2	102	23.8	
CEA*							0.084
–	208	14.3	157	15.4	51	11.9	
+	1242	85.7	864	84.6	378	88.1	
**Reginal nodes examined**							0.723
0	921	63.5	639	62.6	282	65.7	
1–12	129	8.9	93	9.1	36	8.4	
13–25	311	21.4	224	21.9	87	20.3	
26–76	89	6.1	65	6.4	24	5.6	
AJCC N stage*							0.582
N0	580	40.0	401	39.3	179	41.7	
N1	586	40.4	414	40.5	172	40.1	
N2	284	19.6	206	20.2	78	18.2	
**Primary tumor size, (cm)**							0.683
< 3	875	60.3	609	59.6	266	62.0	
3 ≤ < 5	417	28.8	300	29.4	117	27.3	
≥ 5	158	10.9	112	11.0	46	10.7	

*Descending colon included descending colon and sigmoid colon.

Extra-lung metastasis: Colorectal cancer patients involving lung metastases and one or more other distant metastases (mainly bone, brain, and liver metastases).

*CEA* carcinoembryonic antigen; *AJCC* American Joint Commission on Cancer.

### Survival analysis and independent prognostic factors in CRC patients with synchronous LM

**Figure 2:**
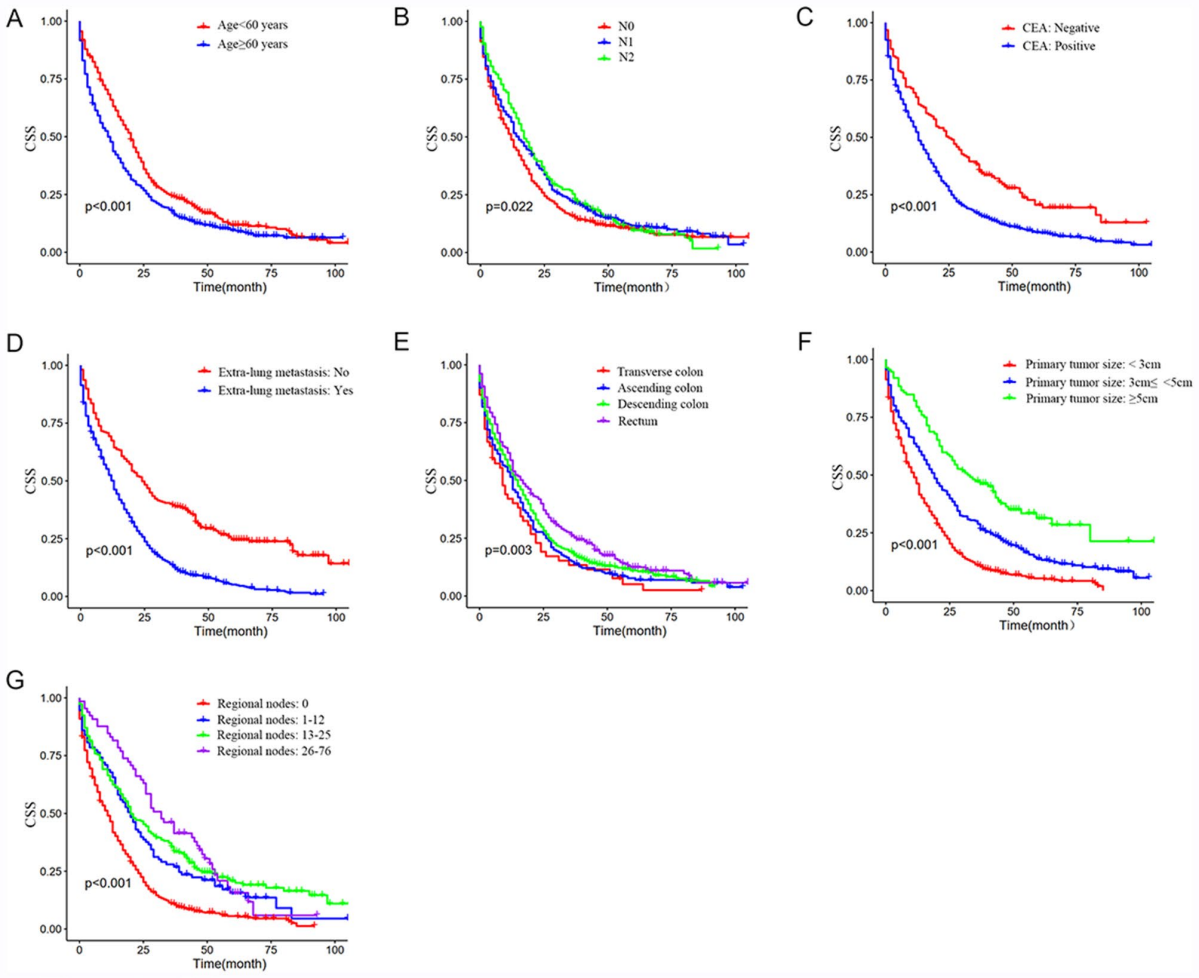
Kaplan–Meier CSS curves stratifying patients by different prognostic variables with *P* < 0.05. (**A**) Kaplan–Meier CSS curve according to age, (**B**) AJCC N stage. and (**C**) CEA level. (**D**) Kaplan–Meier CSS curve according to extra-lung metastasis and (**E**) primary tumor site. (**F**) Kaplan–Meier CSS curve according to primary tumor size and (**G**) the regional nodes examined.

The survival curves for different variables were generated using the Kaplan–Meier method and were compared using the log-rank test. Seven variables, including age, AJCC N stage, CEA level, extra-lung metastasis, primary tumor site, primary tumor size, and regional nodes examined, were associated with CSS (*P* < 0.05) (Table 2). The Kaplan–Meier curves for these factors are shown in Fig. 2. Next, we performed multivariate Cox regression analysis among the seven variables above, and found age (hazard ratio [HR ] = 1.39; 95% confidence interval [CI] = 1.211–1.595; *P* < 0.001); CEA level (HR = 1.426; 95% CI = 1.170–1.738; *P* < 0.001); extra-LM (HR = 1.752; 95% CI = 1.478–2.076; *P* < 0.001); primary tumor site (transverse colon: reference; ascending colon :HR = 0.899; 95% CI = 0.656–1.233; descending colon :HR = 0.824; 95% CI = 0.614–1.106; rectum: HR = 0.681; 95% CI = 0.500–0.928, *P* = 0.014), primary tumor size (< 33 cm: reference; ≤ 3 to < 5 cm: HR = 0.955; 95% CI = 0.660–1.382; ≥ 5 cm: HR = 0.613; 95% CI = 0.424–0.888, *P* = 0.002), and examined reginal nodes examined (< 12: reference; 1–12: HR = 0.739; 95% CI = 0.497–1.098; 13–25: HR = 0.582; 95% CI = 0.398–0.851; 26–76: HR = 0.502; 95% CI = 0.319–0.790, *P* = 0.010) were significantly corelated with CSS and were also defined as independent prognostic factors of CRC patients with synchronous LM (Table 3).

**Table 2:** Univariable analysis in training cohort.

Characteristics	Patients with LM (N = 1021)	%	*P-*value
**Sex**			0.401
Male	563	55.1	
Female	458	44.9	
**Age, years**			0.000
< 60	387	37.9	
≥ 60	634	62.1	
**Year of diagnosis**			0.207
2010	118	11.6	
2011	172	16.8	
2012	172	16.8	
2013	180	17.6	
2014	167	16.4	
2015	212	20.8	
**Race**			0.250
Black	185	18.1	
White	836	81.9	
**Primary tumor site**			0.003
Transverse colon	54	5.3	
Ascending colon	182	17.8	
Descending colon*	469	45.9	
Rectum	316	31.0	
**Pathological type**			0.272
Adenocarcinoma in adenoma	93	9.1	
Adenocarcinoma	880	86.2	
Mucinous adenocarcinoma	41	4.0	
Signet-ring cell carcinoma	7	0.7	
**Primary tumor resection**			0.104
Yes	8	0.8	
No	1013	99.2	
Extra-lung metastasis*			0.000
Yes	754	73.8	
No	267	26.2	
CEA*			0.000
−	157	15.4	
+	864	84.6	
**Reginal nodes examined**			0.000
0	639	62.6	
1–12	93	9.1	
13–25	224	21.9	
26–76	65	6.4	
AJCC N stage*			0.022
N0	401	39.3	
N1	414	40.5	
N2	206	20.2	
**Primary tumor size, cm**			0.000
< 3	609	59.6	
≥ 3 to < 5	300	29.4	
≥ 5	112	11.0	

*Descending colon included descending colon and sigmoid colon.

Extra-lung metastasis: Colorectal cancer patients involving lung metastases and one or more other distant metastases (mainly bone, brain, and liver metastases).

*CEA* carcinoembryonic antigen; *AJCC* American Joint Commission on Cancer.

**Table 3:** Multivariable Cox model of CSS after forward selection of variables with P < 0.05 in univariate Kaplan–Meier analyses.

Characteristics	Hazard Ratio	95% Confidence Interval	*P*-value
**Age, years**			0.000
< 60	1		
≥ 60	1.390	1.211–1.595	
**Primary tumor site**			0.014
Transverse colon	1		
Ascending colon	0.899	0.656–1.233	0.510
Descending colon*	0.824	0.614–1.106	0.197
Rectum	0.681	0.500–0.928	0.015
Extra-lung metastasis*			0.000
No	1		
Yes	1.752	1.478–2.076	
CEA*			0.000
−	1		
+	1.426	1.170–1.738	
**Reginal nodes examined**			0.010
0	1		
1–12	0.739	0.497–1.098	0.134
13–25	0.582	0.398–0.851	0.005
26–76	0.502	0.319–0.790	0.003
AJCC N stage*			0.580
N0	1		
N1	1.011	0.867–1.178	0.889
N2	1.109	0.902–1.363	0.326
**Primary tumor size, cm**			0.002
< 3	1		
≥ 3 to < 5	0.955	0.660–1.382	0.807
≥ 5	0.613	0.424–0.888	0.010

*Descending colon included descending colon and sigmoid colon.

Extra-lung metastasis: Colorectal cancer patients involving lung metastases and one or more other distant metastases (mainly bone, brain, and liver metastases).

*CEA* Carcinoembryonic antigen; *AJCC* American Joint Commission on Cancer.

### Development and assessment of predictive nomogram

**Figure 3:**
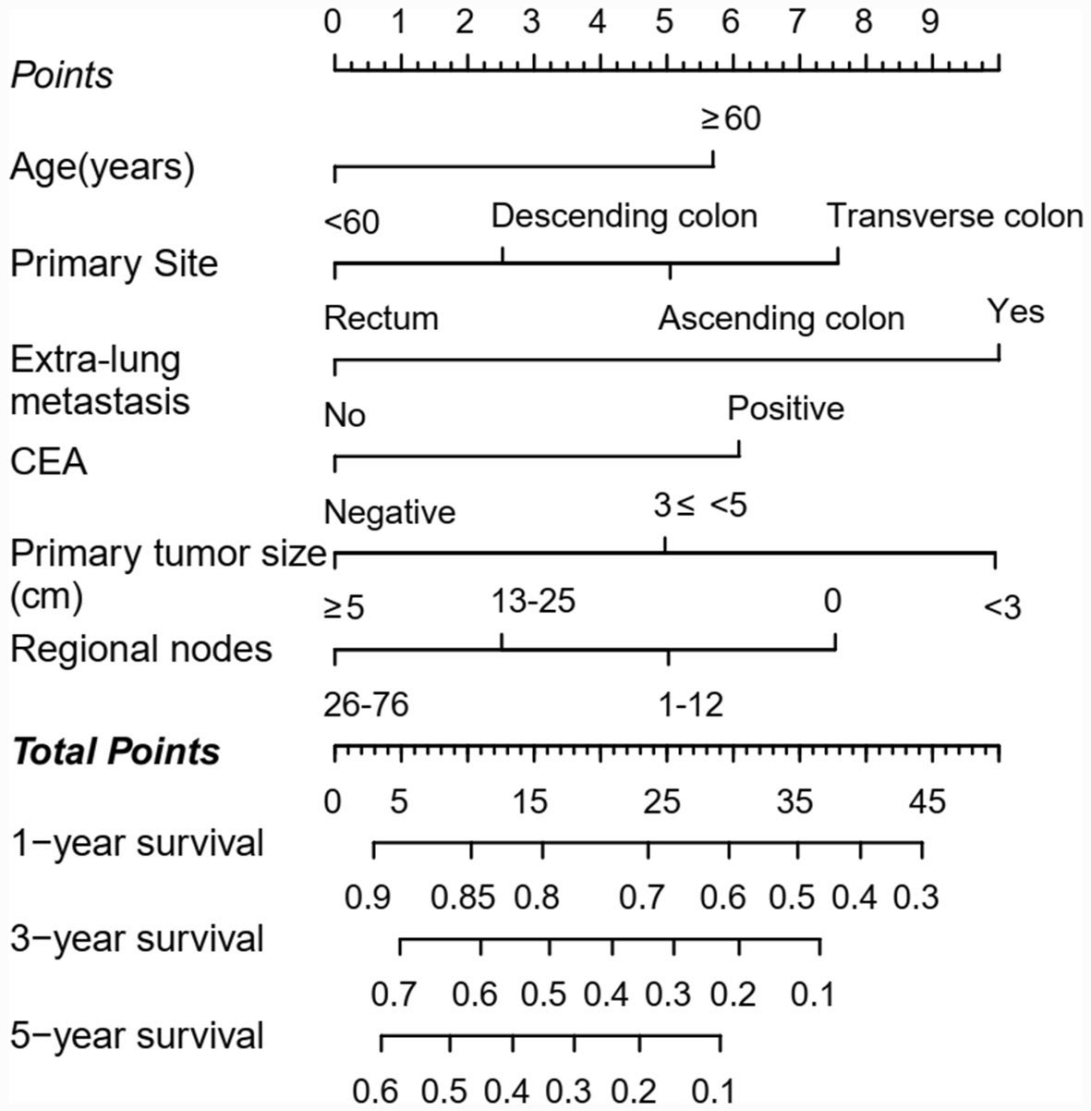
Nomogram for predicting the 1-, 3-, and 5-year CSS. The total score was obtained according to the value of each indicator (age, primary site, extra-lung metastasis, CEA, primary tumor size and regional nodes examined), and the 1-, 3-, and 5-year CSS corresponding to the total score was the predicted rate by nomogram.

**Figure 4:**
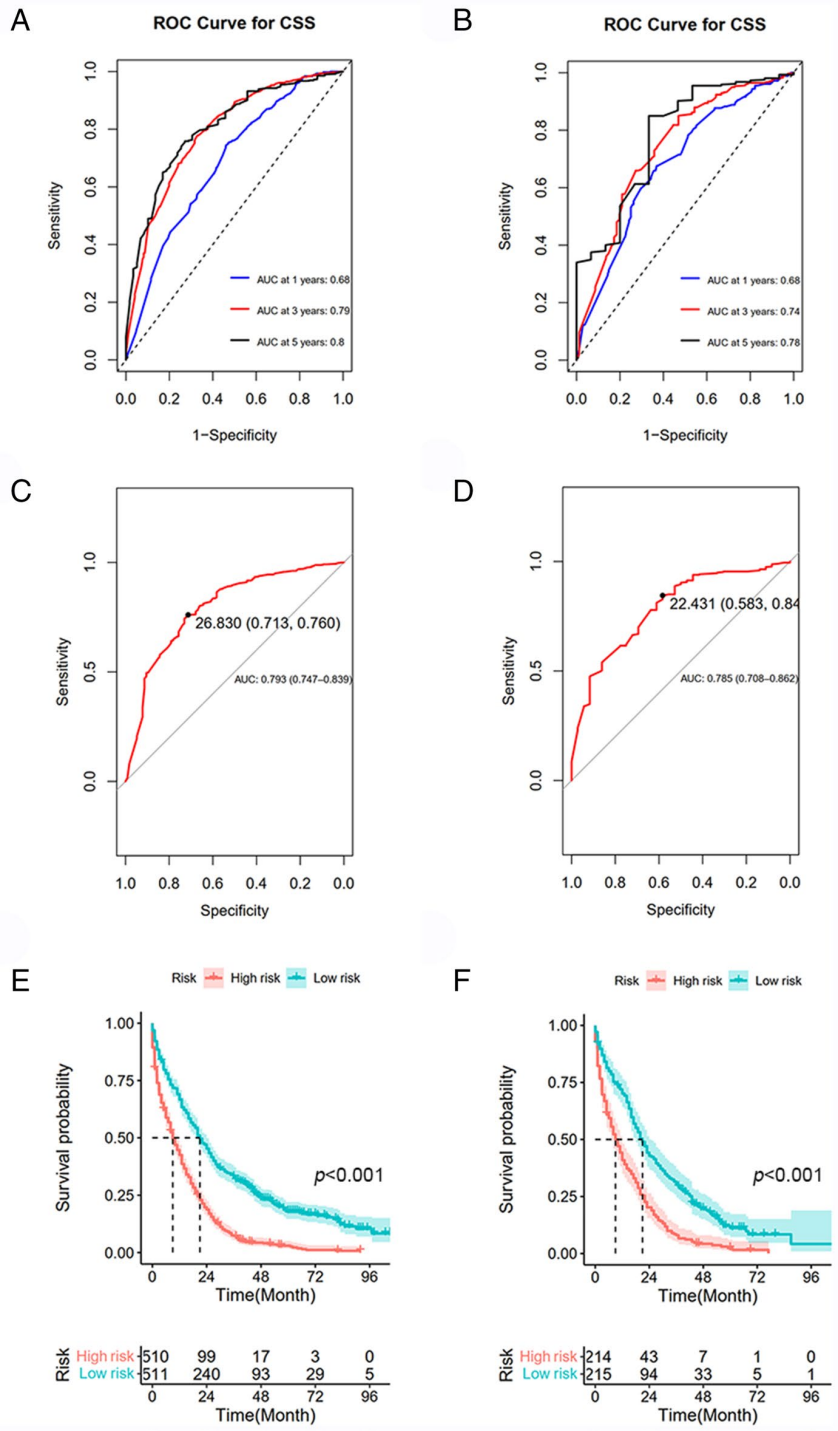
ROC curves based on nomogram and Kaplan–Meier curve of CSS in the training cohort and validation cohort. (**A**) ROC curve and AUC of the nomogram to predict 1-, 3- and 5-year CSS in the training cohort and (**B**) in the validation cohort. (**C**) ROC curve and AUC of nomogram in the training cohort and (**D**) in the validation cohort. (**E**) Kaplan–Meier curve of CSS in the training cohort and (**F**) in the validation cohort. Low- and high-risk groups were divided based on the median total points.

We developed a predictive nomogram containing variables including age,primary site, extra-lung metastasis, CEA, primary tumor size and regional nodes examined, which were demonstrated to be statistically significant in multivariate analysis (Fig. 3). To confirm that the nomogram prediction model had higher efficacy in predicting the prognosis of patients with CRC with synchronous LM, time-dependent ROC analyzes were performed at 1, 3, and 5 years. The AUC of CSS at 1, 3, and 5 years was 0.68 (95% CI = 0.65–0.71), 0.79 (95% CI = 0.75–0.83), and 0.80 (95% CI = 0.75–0.86), respectively in the training cohort (Fig. 4A); whereas 0.68 (95% CI = 0.63–0.73), and 0.74 (95% CI = 0.68–0.80) and 0.78 (95% CI = 0.66–0.90) in the validation cohort (Fig. 4B). The results demonstrated that the 1-, 3-, and 5-year CSS predictions were good.

### Internal and external validation of the nomogram

The C-index and ROC curves were used to evaluate the discrimination power of the nomogram. For internal validation, the bootstrap-corrected C-index for the nomogram was 0.648. For the predictive survival ability of the nomogram in the external validation cohort, the score for the individual case in the validation cohort was calculated according to the established nomogram and then used in the Cox regression model. C-index for the validation cohort was 0.638. To further clarify the discrimination power of the nomogram. The model had an AUC of 0.793 (95% CI, 0.747–0.839), which showed good discrimination (Fig. 4C). The AUC of the validation cohort was 0.785 (95% CI, 0.708–0.862) which demonstrated the nomogram was well fitted (Fig. 4D).

### Risk stratification based on nomogram scores

To further explore the predictive capacity of the nomogram, the total point of each patient was determined based on the nomogram in both the training and validation cohorts. The median point was 33.56 and 28.52 in the training and validation cohort, respectively. Then, we divided the patients into low- and high-risk groups according to the median points and performed a survival analysis using the Kaplan–Meier method. The mean CSS of the training cohort were 13.97 (95% CI = 12.59–15.36) months and 33.07(95% CI = 30.06–36.08) months in the high- and low-risk groups (*P* < 0.001) (Fig. 4E), and in the validation cohort, the mean CSS of the high- and low-risk groups were 14.36 (95% CI = 12.27–16.45) months and 29.32 (95% CI = 25.36–33.28) months respectively (*P* < 0.001) (Fig. 4F). The above results illustrated that patients in the high-risk group tended to have poorer outcomes than those in the low-risk group, and the nomogram had good distinguishing ability and generalizability.

### Calibration curve analysis of the nomogram

**Figure 5:**
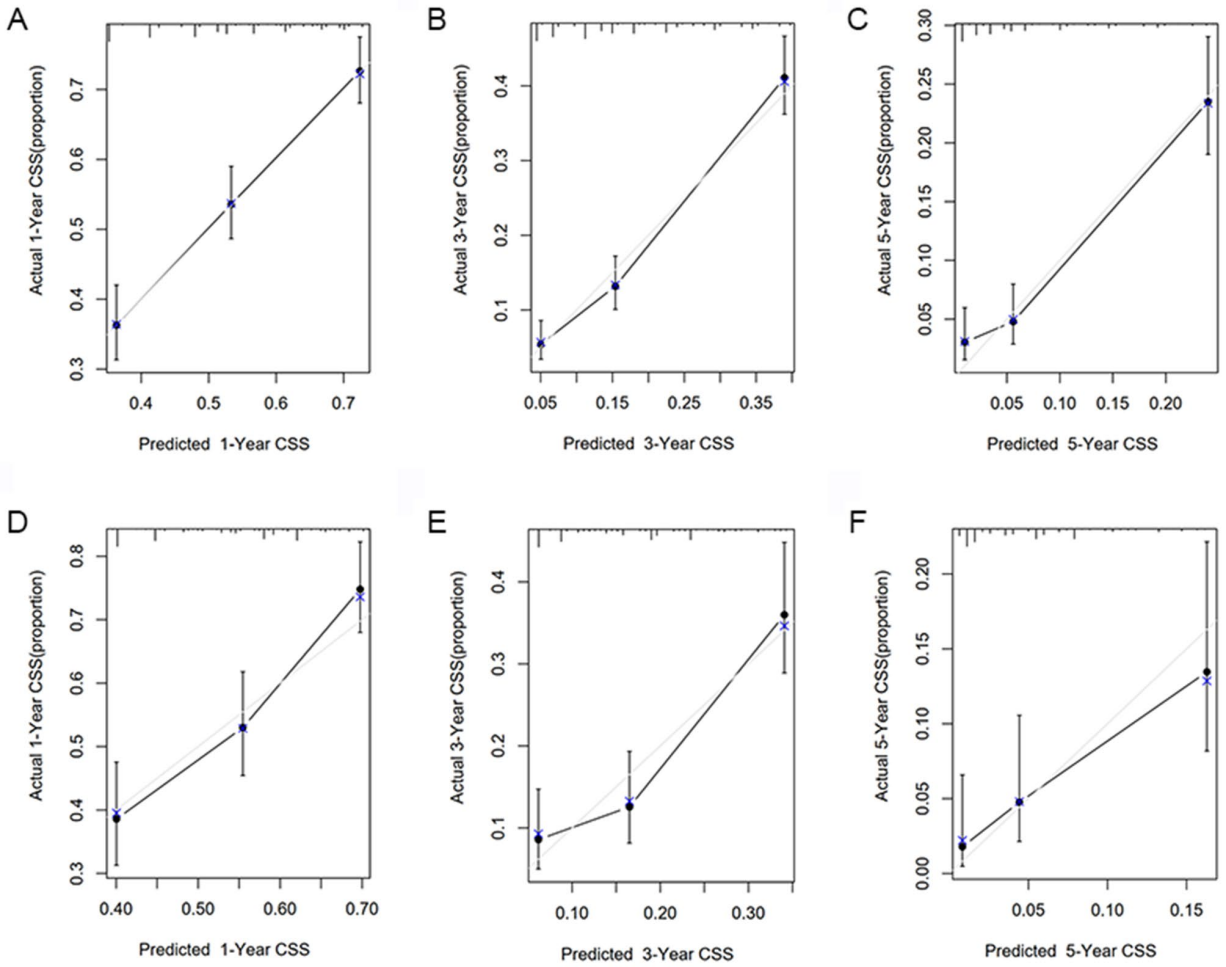
Calibration curves of nomogram in the training and validation cohort. Training cohort: (**A**) 1-year, (**B**) 2-year, and (**C**) 3-year nomogram calibration curves. Validation cohort: (**D**) 1-year, (**E**) 2-year, and (**F**) 3-year nomogram calibration curve.

The bootstrapping method (1000 repetitions) was used and a calibration curve was illustrated in Fig. 5. There were no obvious deviations between the model predicted risk and the actually observed risk curves (Fig. 5A–C), meaning good agreement between observed and predicted 1-, 3-, and 5-year CSS predicted by the nomogram in the training cohort. We further validated the model in the validation cohort using the same method (Fig. 5D–F) and good calibration was observed.

## Discussion

This study focused on the prediction of the 1-, 3-, and 5-year CSS of CRC patients with synchronous LM. From the perspective of predicting indicators, age, CEA levels, extra-LM, primary tumor site, primary tumor size, and regional nodes examined were defined as independent prognostic factors of stage IV CRC patients with synchronous LM. A nomogram based on the aforementioned variables was constructed to forecast the 1-, 3-, and 5-year CSS, and the discrimination ability was estimated by calibration and discrimination in both training and validation cohorts. The calibration curve of 1-, 3-, and 5-year CSS in the training cohort showed favorable agreement between the predicted and actual observed probabilities, similarly, the validation cohort also showed optimal calibration in the 1-, 3-, and 5-year CSS, which supported the repeatability as well as reliability of the constructed model. Then, we stratified CRC patients with LM into high-risk and low-risk groups according to median individual points. This stratification indicated that patients in the high-risk group had poorer CSS, therefore, more intensive follow-up and more comprehensive treatment should be considered. Both the C-index and AUC values revealed the good discriminatory capacity of the nomogram: the C-index of the training and validation groups were 0.648 and 0.638, respectively, and the AUC values were 0.793 and 0.785, respectively. Furthermore, the clinical parameters to be input into the nomogram are easy to obtain from the patient’s clinical records, making it a simple tool to use. Overall, our nomogram had a good ability to predict CSS and could be applied as a convenient tool to predict 1-, 3-, and 5-year CSS of CRC with synchronous LM.

The present study revealed that the prognosis of CRC patients with synchronous LM was better among individuals < 60 years of age, which was consistent with previous studies^20,21^ reporting that age was an independent prognostic factor for CRC patients with synchronous LM and suggested that in an increasingly aging population, the functional status of patients should be taken into account. Furthermore, many studies have reported that the primary tumor site could be a prognostic factor for metastasis CRC^22–24^. A proposed explanation is that the colon and rectum present differences in the microbiome composition, chromosomal, and molecular characteristics^25^. Therefore, colorectal tumors with different sites have different capacities for metastasis. This study shows that the primary tumor site could be prognostic in CRC with LM. We showed that different CSS could be predicted for patients with tumors at the right colon, transverse colon, descending colon, and rectum. The primary tumor in the transverse colon for CRC with LM had worse CSS. A high level of CEA was associated with CRC. Preoperative CEA levels were important in determining diagnosis and prognosis and were widely used in clinical practice^20,26,27^, and during the post-resection follow-up period, the CEA level was an important indicator to detect local recurrence and distant metastases after surgery in CRC patients. However, it remains unknown whether CEA was an independent factor in CRC survival with LM. A study evaluating synchronous CRC reported^26^ that patients with multiple colorectal tumors were more likely to express high levels of CEA, Unfortunately, it did not explore the relationship between CEA and the prognosis of synchronous colorectal carcinoma. Although, our study demonstrated that elevation of the CEA level could predict the prognosis of patients with CRC with synchronous LM.In this study, tumor size < 3 cm was shown to be associated with poor CSS. This result was inconsistent with previous research^28,29^, which showed that tumor size ≥ 5 cm was an independent risk factor for a worse prognosis. However, in the clinic, there may be cases of locally advanced CRC with small primary tumors and small colorectal tumors do not always correlate with early-stage disease. Furthermore, CRC patients with synchronous LM were not the main target investigated in previous studies. Our research indicated that for CRC patients with synchronous LM, smaller tumors tended to have stronger invasiveness and metastatic capacity. Patients with a tumor size < 3 cm may have a poorer prognosis, but further studies are needed. In our study, extra-LM lesions were also identified as a prognostic factor. The presence of extra-LM is associated with a more aggressive primary tumor, on the other hand, extra-LM could influence a patient’s organ function, aggravating symptoms, therefore, the extra-LM may represent a negative factor in the CSS of the patients. Cancer cells in regional lymph nodes^30^ are often associated with reduced survival. Radical surgery including lymphadenectomy of CRC is a decisive factor for prognosis and is necessary for the therapeutic staging of the patient, the minimum number of 12 examined lymph nodes has been accepted in clinics since 1990^31^. We found that the identification of fewer than 12 regional nodes was associated with a worse CSS, which was consistent with many studies^20,32^. In particular, the role of primary tumor resection in CRC patients with distant metastases remains controversial. A previous study^33^ reported better outcomes in patients with CRC LM undergoing lung metastasectomy, and also achieved remarkable improvement in prognosis. But several studies^34,35^ have also suggested that surgical resection should not be performed if all known tumors could not be completely removed (R0 resection), because it could not provide survival benefits for CRC patients with metastatic diseases. A recent study^36^ also found that prognosis was similar for those who underwent lung metastasectomy and those who did not. Our study showed that primary surgery cannot comprehensively improve CSS for patients with CRC with synchronous LM. The explanation may be due to the advancement of palliative treatment including chemotherapy and molecular targeting treatment (i.e. FOLFOX or FOLFIRI combined with molecularly targeted drugs), which has improved the prognosis of CRC patients with LM and will continue to improve. However, this needs to be further studied as only 0.8% of the patients in the study had completed primary surgery, which means that a selection bias cannot be avoided, furthermore, detailed treatment information including chemotherapy was not recorded in the SEER database; thus, further studies should include more cases with lung metastasectomy and treatment information.

This large-scale study revealed the clinical characteristics, risk, and prognostic factors for CRC patients with synchronous LM. However, this study still has some limitations. First, this was a retrospective study that included patients from the United States. A future external validation involving patients from other countries is needed to evaluate the generalizability of this nomogram. Moreover, as lung metastasis was the aim of this study, extra-lung metastasis including bone, brain, and liver metastases was not discussed individually while they could influence the evaluation for the CSS. Therefore, Cox regression model might not be the best method to analysis the CSS and a more suitable competing risk model would be applied in the next study, however, the result of this study was still helpful in our future study to further research the CSS influence of other coexisted metastasis for CRC patients with lung metastasis. Also, the 1-year AUC value of the nomogram based on both the training and validation group was 0.68, which suggests the reliability of the 1-year CSS prediction needs to be enhanced. In addition, some patients who received surgery solely targeting their colorectal tumors were not further extracted from this study. Further studies were needed to clarify the necessity of palliative surgery without lung metastasectomy. Finally, this study did not investigate specific treatment options, because detailed treatment information, including radiation therapy, was not recorded in the SEER cohort. In summary, this nomogram was based on the SEER database and made full use of its indicators. The incorporation of other factors, such as additional biomarkers, will improve this model. Despite these limitations, this nomogram remains a good risk model and could be applied to predict the prognosis of stage IV CRC patients with synchronous LM.

In summary, currently, patients with stage I to III tumors who undergo primary surgery are predicted to have a good prognosis; whereas, the prognosis for stage IV CRC patients with LM remains poor. Although these patients only account for a small proportion of CRC patients, greater attention should be placed on their prognosis. In this study, we constructed and validated a predictive nomogram for the CSS of CRC patients with synchronous LM, which could be used to accurately evaluate the 1-, 3-, and 5-year CSS of stage IV CRC patients with synchronous LM and help distinguish high-risk patients who may require more aggressive treatment and follow-up strategies.

## Data Availability

Publicly available datasets were analyzed in this study. This data can be found here: https://seer.cancer.gov/.

## Abbreviations

CRC: Colorectal cancer
LM: Lung metastases
CSS: Cancer-specific survival
SEER: Surveillance, epidemiology, and end results database
C-index: Concordance index
ROC: Receiver operating characteristic curves
CEA: Carcinoembryonic antigen
AJCC: American Joint Commission on Cancer
TNM: Tumor-node-metastasis
HR: Hazard ratio
CI: Confidence interval
